# Editorial: Live cell imaging: Cell and developmental research bridging education, optical engineering, industry, software, shared facilities

**DOI:** 10.3389/fcell.2023.1191235

**Published:** 2023-06-15

**Authors:** Michelle S. Itano, Abhishek Kumar, Paul S. Maddox

**Affiliations:** ^1^ University of North Carolina at Chapel Hill, Chapel Hill, NC, United States; ^2^ Marine Biological Laboratory (MBL), Woods Hole, MA, United States

**Keywords:** imaging, optics and imaging, collaboration, technology development, fluorescence

Optical imaging is one of the original technological pillars of biomedical research, spanning centuries of discovery. Drive for biological understanding has fueled significant progress in imaging tool development, ranging from illumination sources, detectors, and mechanical stages, to novel optical components and analysis strategies. The number and variety of optical imaging tools is now so vast that in some cases cutting-edge technologies require specialized training across a wide set of skills. Considering the exponential growth in imaging tools, fruitful and conducive partnerships between academic researchers, commercial vendors, and instrument facilitators, such as core facility managers and staff, are more important than ever.

Advancement: Optical imaging has advanced at an unprecedented speed in the past decade fueled by innovation on multiple fronts. This spur in tool availability has generated multiple choices for researchers from a wide range of specialized, customized techniques to turnkey commercial systems (Weber and Huisken, Gibbs et al., Hobson et al., Varady and Distel). Microscope startup companies play an important role in this ecosystem and facilitate researchers’ rapid ability to design a system or range of systems best suited to their specific experimental needs and preferences. Additionally, many laboratories, researchers, and companies developing such technologies are sharing their results and their processes openly and helping advance the science of the broader imaging community (Weber and Huisken, Smith et al., Katunin et al., Alghamdi et al., Marston et al., Bostock et al.). We anticipate that tool development will keep advancing and cross-disciplinary collaboration will increasingly become a necessity, which is a positive sign for the biomedical sciences and scientific community (Chandris et al.).

Access: Although the scientific community has made tremendous advances on all fronts of optical imaging, the Frontier is moving ever forward and translating this technology to the general, non-technical, biomedical community is ever challenging. Many researchers, universities, and research funders are taking an active interest in opening the door to enable the translation of these technologies for all to use. Innovation on the “distribution” side is also ongoing with microscope companies setting up centers where researchers can access advanced tools at a moderate cost. This approach circumnavigates the requirement to hire expert trainers and to make a permanent, significant capital investment. Making advanced tools along with expert help accessible is still a challenge and it requires investment from federal organizations, private foundations, and an active effort from universities and scientists. Additionally, various professional and community-building societies such as BINA (BioImaging North America), Global Bioimaging, Association of Biomolecular Resource Facilities (ABRF), etc., are advancing and advocating access for researchers. Optical microscopy training courses and workshops (e.g., those offered by the Marine Biological Laboratory, Janelia Research Campus, and Cold Spring Harbor Laboratory) are critical in training scientists across all levels of the research collective who share common interests in using imaging science in their research. These courses serve as the backbone of the training ecosystems required to best use advanced imaging tools in the future.

Avenues: The field of optical imaging offers avenues for collaboration, contribution, and complementation. Three broad areas that can benefit from interdisciplinary collaborations, for example, are a) data handling and image processing/analysis, b) information and protocols for sample preparation and mounting and c) sharing microscope parts and design details for advanced tools so that scientists can adopt such technologies during the pre-commercialization phase. There is a growing need to foster collaborations more efficiently and provide avenues to further enhance resource sharing for optimal use of optical microscopy and associated image analysis approaches. Effective collaborations require investment from a broad range of individuals representing perspectives from institutional administrators, trainers, developers, investors, and those actively collecting and analyzing data, including those who may be new to the imaging field. In order for these initiatives that foster increased collaboration to be successful, they will require multiple years (4–5 years, similar to the length of most research program grants) of commitment from a dedicated group of individuals and financial investment in order to support the prioritization of these activities. Currently, the main limitation to growth in this arena is the lack of funding opportunities for a time period that would allow for broad and sustainable impact.

We hope you enjoy and utilize this special edition of research articles, tutorial perspectives and methodological protocols that are advancing the field of imaging science. It is our intent to be a step toward embracing the complexities as we move into a new era accelerating both innovation and access in biomedical imaging.

